# GABA and l-theanine mixture decreases sleep latency and improves NREM sleep

**DOI:** 10.1080/13880209.2018.1557698

**Published:** 2019-02-01

**Authors:** Suhyeon Kim, Kyungae Jo, Ki-Bae Hong, Sung Hee Han, Hyung Joo Suh

**Affiliations:** a Department of Integrated Biomedical and Life Sciences, Graduate School, Korea University, Seoul, Republic of Korea;; b Department of Biological Sciences and Environmental Sciences Program, Southern Illinois University-Edwardsville, Edwardsville, IL, USA;; c BK21 Plus, College of Health Science, Korea University, Seoul, Republic of Korea;; d Department of Public Health Sciences, Graduate School, Seoul, Republic of Korea

**Keywords:** γ-Aminobutyric acid, insomnia, pentobarbital-induced sleep test, electroencephalography

## Abstract

**Context:** γ-Aminobutyric acid (GABA) is the main inhibitory neurotransmitter and it is well established that activation of GABA_A_ receptors favours sleep. l-Theanine, a naturally occurring amino acid first discovered in green tea, is a well-known anti-anxiety supplement with proven relaxation benefits.

**Objective:** This study investigated the potential synergistic sleep enhancement effect of GABA/l-theanine mixture.

**Materials and methods:** Pentobarbital-induced sleep test was applied to find proper concentration for sleep-promoting effect in ICR mice. Electroencephalogram (EEG) analysis was performed to investigate total sleeping time and sleep quality in normal SD rats and caffeine-induced awareness model. Real-time polymerase chain reaction (RT-PCR) was applied to investigate whether the sleep-promoting mechanism of GABA/l-theanine mixture involved transcriptional processes.

**Results:** GABA/l-theanine mixture (100/20 mg/kg) showed a decrease in sleep latency (20.7 and 14.9%) and an increase in sleep duration (87.3 and 26.8%) compared to GABA or theanine alone. GABA/l-theanine mixture led to a significant increase in rapid eye movement (REM) (99.6%) and non-REM (NREM) (20.6%) compared to controls. The use of GABA/l-theanine mixture rather than GABA or l-theanine alone restored to normal levels sleep time and quality in the arousal animal model. The administration of GABA/l-theanine led to increased expression of GABA and the glutamate GluN1 receptor subunit.

**Conclusions:** GABA/l-theanine mixture has a positive synergistic effect on sleep quality and duration as compared to the GABA or l-theanine alone. The increase in GABA receptor and GluN1 expression is attributed to the potential neuromodulatory properties of GABA/l-theanine combination, which seems to affect sleep behaviour.

## Introduction

Sleep loss and other related disturbances pose an important health problem, as they can lead to significant functional impairments. Sleep disturbances can affect daily life considerably and reduce the quality of life. The importance of a good night’s sleep is well-established, nevertheless many people suffering from sleep disorders prefer not to use hypnotic drugs, despite providing effective symptomatic relief.

Hypnotic drugs, such as benzodiazepine analogues, zolpidem and doxepin, can cause unexpected side effects and can lead to drug resistance and dependence (Longo and Johnson 2000; Victorri-Vigneau et al. 2007; Lichstein et al. 2013). These types of sleeping drugs are not suitable for the treatment of temporary anxiety or sleep disturbances due to the observed drug resistance and dependence that has been associated with their long-term use (Oh et al. 2010). However, herbal remedies have been reported as effective and with a relatively low side effect risk for the treatment of insomnia (Wheatley 2005). Therefore, it is necessary to develop new bioactive substances derived from natural sources that present with similar efficacy but fewer side effects than hypnotic drugs, for the successful treatment of sleep-related disturbances.

γ-Glutamylethylamine, also known as l-theanine, and γ-aminobutyric acid (GABA) are known agents for improving sleep disturbances (Khan et al. 2018). GABA is a non-proteinogenic amino acid and is the main inhibitory neurotransmitter in the mammalian brain. Hence GABA_A_ receptors are a primary target in the search for natural anxiolytic compounds or sedatives (Khom et al. 2007; Trauner et al. 2008). There is an increasing interest in investigating the effect of GABA-mediated inhibitory neurotransmission, in respect to its potential benefit on counteracting sleep disruption caused by various conditions, such as stress, diseases and caffeine intake, etc. (Wong et al. 2003). Therefore, GABA is widely used in functional food and pharmaceutical industries, and various researches have been investigated for biosynthesis and their efficacy as metabolites of plants and microorganisms produced by the decarboxylation of glutamic acid (Coda et al. 2010; Dhakal et al. 2012; Yang et al. 2018) l-Theanine, an amino acid exclusively found in tea leaves, composes only 1–2% (w/w) of the weight of dried tea leaves (Graham 1992) and is chemically or biologically synthesized for use as an active ingredient that induces sedation (Juneja et al. 1999; Yan et al. 2003). There are several reports indicating that l-theanine exerts neuroprotective effects (Kim et al. 2009), modulates neurotransmitter activity (Kakuda 2011) and reduces psychological stress (Kimura et al. 2007) and sleep disturbances (Jang et al. 2012). Nathan et al. (2006) also reported that l-theanine intake increases serotonin, dopamine and GABA levels in the brain.

In recent years, there have been numerous ‘relaxation beverages’ available on the market containing relaxation-inducing nutraceuticals, such as valerian, l-theanine, GABA, 5-hydroxytryptophan (5-HTP) and the sleep-aid, melatonin. Therefore, the combination of GABA and l-theanine may synergistically promote symptomatic relief for sleep disorders, despite the scarce experimental data supporting this process. The purpose of this study was to investigate whether the effect of GABA/l-theanine mixture on sleep disturbances is greater than GABA or l-theanine alone and to determine the most effective dosing combination.

## Materials and methods

### Materials

GABA (90.8%) and l-theanine (99.3%) was supplied by Neo Cremar Co. Ltd (Seoul, Korea) and BTC Co. Ltd (Ansan, Korea), respectively. Caffeine was purchased from Sigma-Aldrich (St. Louis, MO) and pentobarbital sodium was purchased from Hypharm. Co. Ltd. (Gyeonggi-do, Korea). All other reagents were purchased at the highest commercial grade available.

### Animals

Male ICR mice (4 weeks old, 18–20 g) and Sprague-Dawley (SD) rats (8 weeks old, 160–180 g) were purchased from Orient Bio (Orient Bio Inc., Seongnam, Korea). All animals were caged at 22 ± 2 °C and 55 ± 5% humidity with a 12 h light/dark cycle. Normal pellet diet and water were freely provided. Rodents were acclimatized for at least one week before starting pentobarbital-induced sleep testing and electroencephalography (EEG) analysis. The ages of the animals used in this study were to ensure the functional integrity of the brain and central nervous system, which usually does not occur in older animals, which normally have degraded morphological and functional characteristics (Verdú et al. 2000). In this study, all animal experimental protocols were approved by the Korean University Animal Care Committee (KUIACUC-2017-49, Seoul, Korea).

### Pentobarbital-induced sleep test

Pentobarbital-induced sleep was performed according to previously established methods with slight modifications (Yang et al. 2013). Sodium pentobarbital (42 mg/kg) was administered intraperitoneally in each mouse 40 min after oral administration of GABA, l-theanine or both (GABA/l-theanine). The time elapsed from compound administration to the loss of righting reflex (sleep latency) and the time from the loss of righting reflex to its return (sleep duration) were measured in seconds. Mice that did not sleep 15 min after the injection were excluded from the experiment.

### Electrophysiological analysis

Male SD rats were anesthetized with 2% isoflurane (Troikaa Pharmaceutical Ltd., Gujarat, India), using a gas anesthesia mask in a stereotaxic instrument frame (Stoelting Inc., Wood Dale, IL). For the EEG recording, EEG screw electrodes were implanted into the cortex, striatum and hippocampus, as previously described (Hong et al. 2016). All rats received antibiotics and were kept individually in cages under a temperature-controlled facility with water and food. The rats were randomly divided into control and treatment groups at 7 days after recovery. Experiments were conducted from 10 am to 5 pm for 9 days. GABA, l-theanine or GABA/l-theanine mixture was orally administered 1 h before EEG signal analysis. EEG signals were amplified, filtered (0.5–30 Hz), recorded and stored using Iox2 (version. 2.8.0.13, emka Technologies, Paris, France). EEG spectra were analyzed in 1 Hz frequency bins and standard frequency bands (β: 13–30 Hz; α: 8–13 Hz; θ: 4–8 Hz; δ: 0.5–4 Hz). After the EEG recording, fast Fourier transform (FFT) was performed every 2 sec. Based on the FFT average data obtained at 10-sec intervals in the range of 0–30 Hz, the ecgAUTO3 program (version. 3.3.0.20, emka Technologies) was used to calculate the awake and sleep time. Caffeine (10 mg/kg) was used to induce the arousal condition before the experiments.

### Quantification of receptor mRNA levels

Total RNA was extracted from mouse brains using TRIzol® (Invitrogen, CA), while genomic DNA was removed using Direct-zolTM RNA Miniprep (ZYMO Research, CA) according to the manufacturer’s protocol. Quality-controlled RNA (1 μg) was reverse transcribed using SuperScript® III Reverse Transcriptase (Invitrogen) with oligo d(T) as the primer. The generated cDNA was subjected to quantitative real-time PCR (qRT-PCR) using a Power Taqman PCR Master Mix kit (Applied Biosystems, Foster City, CA). For qRT-PCR, cycling conditions were 50 °C for 2 min, 95 °C for 10 min, followed by 40 cycles at 95 °C for 15 s and 60 °C for 1 min. Quantitative analysis was conducted using StepOne plus Software version 2.0 (Applied Biosystems, Inc., Foster City, CA). The endogenous housekeeping gene, GAPDH (NM_008084.2), was used for result normalization. Information for the target genes used for qRT-PCR is as follows: GABA_A_ receptor (NM_008076.3), GABA_B_ receptor 1 (NM_01 9439.3), GABA_B_ receptor 2 (NM_001081141.1), GluA1 (NM_00111 3325.2), GluN1 (NM_001177656.2) and GluN2A (NM_008170.2).

### Statistical analysis

Testing results were evaluated for statistical differences using SPSS version 12.0 (SPSS, Chicago, IL) by one-way analysis of variance (ANOVA) followed by both Tukey’s multiple comparisons and Bonferroni *post hoc* test. Different letters indicate significant differences (*p* < 0.05) among groups by Tukey’s multiple comparison tests. All data are expressed as the means ± standard error (SE) comparisons between groups, *n* = 8.

## Results

### Effects of GABA and l-theanine on sleep latency and duration in the pentobarbital-induced sleep model

**Figure 1.**
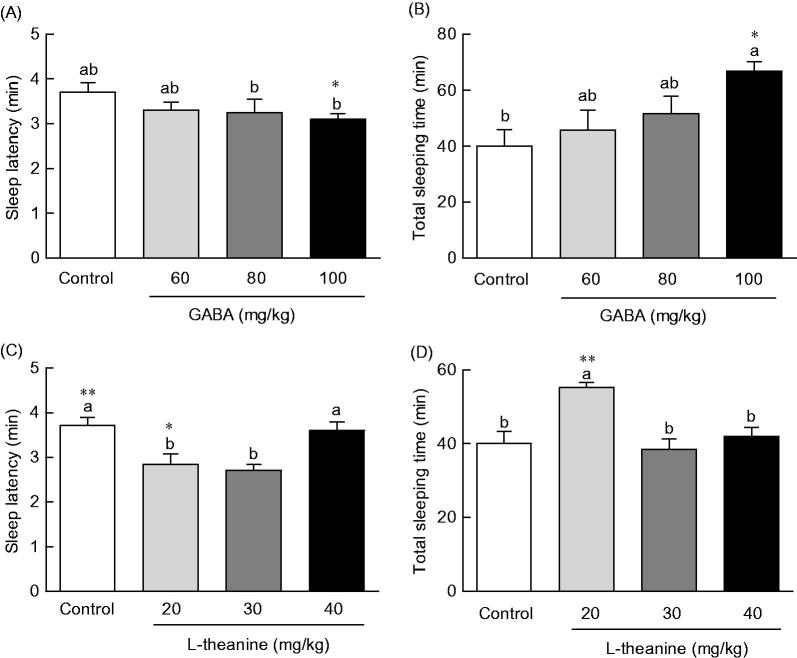
Effects of GABA or l-theanine on sleep latency (A, C) and sleep duration (B, D) in mice administered with a hypnotic dosage of pentobarbital (42 mg/kg, i.p.). Values are presented as the means ± standard error (SE) for each group, *n* = 8. Different letters indicate significant differences (*p* < 0.05) among samples by Tukey’s multiple range test. Symbols indicate significant differences by Bonferroni test, as **p < 0.01, *p < 0.05.

Sleep latency and duration time following GABA or l-theanine administration were measured in the pentobarbital-included sleep model to identify the optimal combination ratio for sleep enhancement (Figure 1). Sleep latency showed a tendency to decrease with increasing GABA concentration and sleep duration to increase with increasing GABA concentration (Figure 1(A,B)). Regarding sleep latency, there was a significant difference (*p* < 0.05) following the administration of 100 mg/kg of GABA (3.1 min), as compared to control (3.7 min), but no significant differences with other GABA concentrations were observed (Figure 1(A)). With regards to sleep duration, there was also a significant difference (*p* < 0.05) following 100 mg/kg of GABA, as compared to control. No significant differences of sleep duration were observed following the administration of any other GABA concentrations (Figure 1(B)). Pentobarbital-induced sleep testing was carried out to assess the potential sleep enhancement effect of l-theanine (Figure 1(C,D)). Following the administration of 20 or 30 mg/kg of l-theanine, sleep latency significantly decreased (2.8 and 2.7 min, respectively), as compared to controls (3.7 min) (*p* < 0.05 and *p* < 0.01, respectively). However, the administration of 40 mg/kg of l-theanine not decreased sleep latency (3.7 min). When compared to controls (39.9 min), total sleep time increased in l-theanine-treated animals at a dose of 20 mg/kg (55.2 min), but not with 30 or 40 mg/kg of l-theanine (Figure 1(D), *p* < 0.01). In summary, administration of 20 mg/kg of l-theanine in mice, resulted in a decrease in sleep latency (23.3%) and an increase in sleep duration time (38.1%).

### Effects of GABA/l-theanine combination on sleep latency and duration in the pentobarbital-induced sleep model

**Figure 2.**
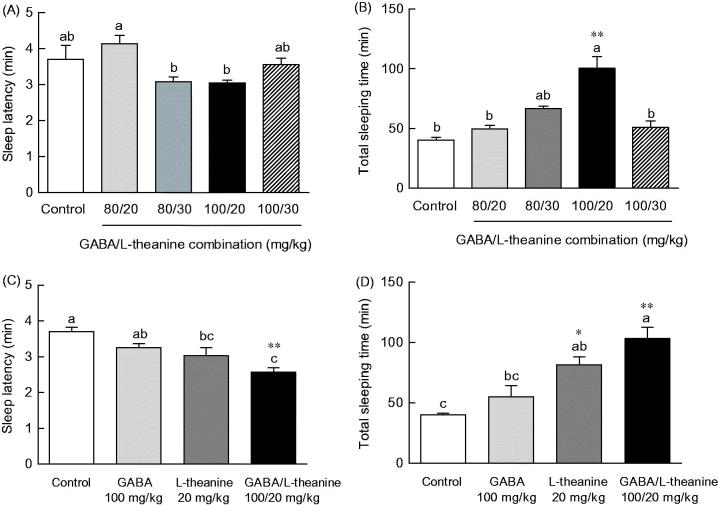
Effects of GABA/l-theanine mixture on sleep latency (A, C) and sleep duration (B, D) in mice administered with a hypnotic dosage of pentobarbital (42 mg/kg, i.p.). Values are presented as the means ± standard error (SE) for each group, *n* = 8. Different letters indicate significant differences (*p* < 0.05) among samples by Tukey’s multiple range test. Symbols indicate significant differences by Bonferroni test, as **p < 0.01, *p < 0.05.

The induced changes in sleep latency and duration time by different ratio combinations of GABA (80 and 100 mg/kg) and l-theanine (20 and 30 mg/kg) were measured (Figure 2). The sleep latency of animals that were administered with either of the GABA/l-theanine dose combinations (80/30 and 100/20 mg/kg) was slightly lower than the control group (Figure 2(A)) (16.8 and 17.7%, respectively). With respect to sleep duration, sleep time showed a tendency increase in all dose combination groups (Figure 2(B)). In particular, the GABA/l-theanine mixture administered at a dose ratio of 100/20 mg/kg, evoked the highest sleep duration increase (100.6 min) (*p* < 0.01).

The effects of the best sleep-promoting GABA/l-theanine dose combination (100/20 mg/kg) were compared to the changes in sleep latency and duration induced by the single administration of GABA or l-theanine (Figure 2(C,D)). The combined use of GABA/l-theanine (100/20 mg/kg) showed a decrease in sleep latency (20.7% and 14.9%, respectively) and an increase in sleep duration (87.3% and 26.8%, respectively) compared to a single administration of GABA (100 mg/kg) or theanine (20 mg/kg). The combined use of GABA/l-theanine showed synergy effects on sleep latency and sleep duration time.

### Effects of GABA and l-theanine mixture on sleep architecture

**Figure 3.**
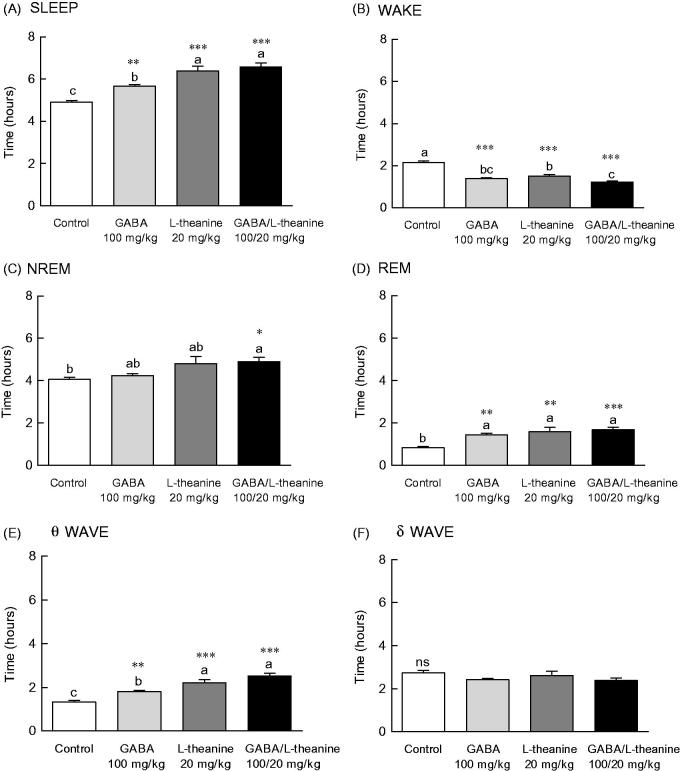
Effects of the GABA, l-theanine, and GABA/l-theanine mixture on sleep quantity and quality. Values are presented as the means ± standard error (SE) for each group, *n* = 8. Different letters indicate significant differences (*p* < 0.05) among samples by Tukey’s multiple range test. Symbols indicate significant differences at ***p < 0.001, **p < 0.01, *p < 0.05 by Bonferroni test. NS: not significant.

EEG parameters were recorded to more accurately confirm the synergistic effect of GABA/l-theanine mixture observed in the pentobarbital-induced sleep model (Figure 3). The changes in sleep time and architecture were measured after a single administration of GABA 100 mg/kg or l-theanine 20 mg/kg and after GABA/l-theanine mixture (100/20 mg/kg). Figure 3 depicts the longest sleep time recorded following the oral administration of GABA/l-theanine. However, no significant difference in sleep time was detected between GABA/l-theanine mixture and l-theanine alone (Figure 3(A)). The most reduced awake time was detected after oral administration of GABA/l-theanine mixture (1.2 h) and was significantly different from control (2.2 h) (Figure 3(B), *p* < 0.001). Single GABA administration (100 mg/kg) significantly increased rapid-eye-movement (REM) sleep time, as compared to the control group (71.5%) (*p* < 0.01), but there was no significant difference in non-REM (NREM) sleep time compared to the control group (Figure 3(C,D)). Single l-theanine administration (20 mg/kg) significantly increased REM time, when compared to controls (88.6%) (*p* < 0.01), but there was no significant difference in NREM sleep time compared to the control group. NREM (20.7%) and REM (99.6%) were also significantly increased than control levels, after GABA/l-theanine mixture was orally administered (Figure 3(C,D), *p* < 0.05 and *p* < 0.001, respectively). The oral administration of GABA/l-theanine mixture improved sleep time and quality, as compared to GABA or l-theanine alone.

The θ wave was significantly increased after GABA (1.8 h) or l-theanine (2.2 h) single infusion, as well as after GABA/l-theanine combined administration (2.5 h) (Figure 3(E)). The δ wave showed a tendency to decrease when GABA or l-theanine was separately administered and after GABA/l-theanine, but there were no significant differences between groups (Figure 3(F)).

### Effects of GABA and l-theanine combination on sleep architecture during three sleep periods

**Figure 4.**
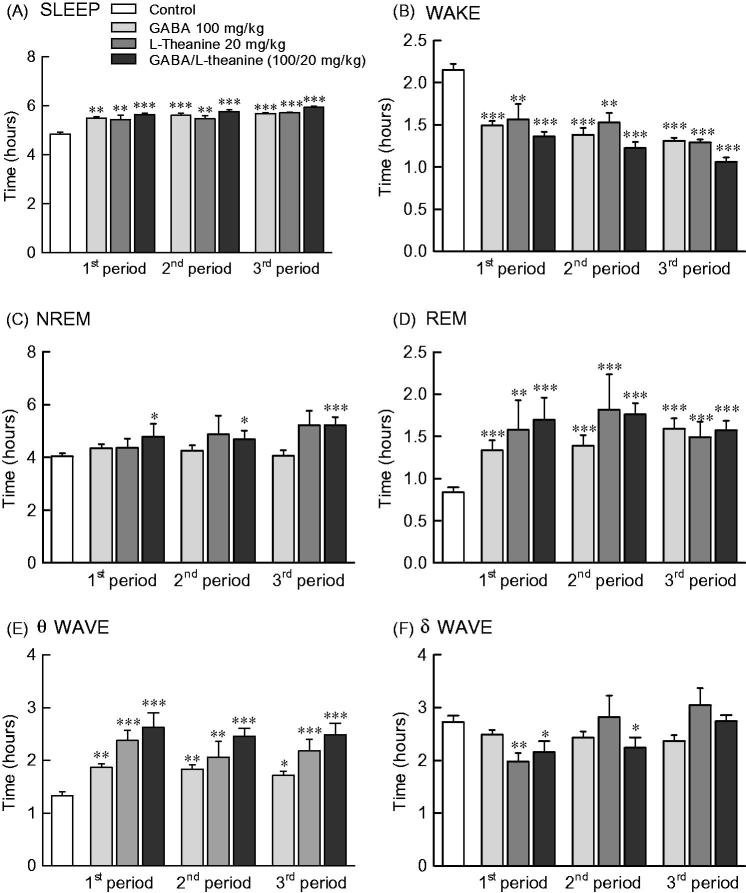
Effects of GABA, l**-**theanine, and GABA/l-theanine mixture on sleep quantity and quality during administration periods. Values are presented as the means ± standard error (SE) for each group, *n* = 8. Symbols indicate significant differences by Bonferroni test, as ***p < 0.001, **p < 0.01, *p < 0.05.

Figure 4 depicts the sleep pattern measured for 9 days divided into 3 periods. Total sleep time increased and awakening time decreased from 1st to 3rd period in all sample groups (Figure 4(A,B)). NREM sleep was significantly increased in the GABA/l-theanine mixture group, as compared to controls during all three periods (Figure 4(C); 1st period: *p* < 0.05, 2nd period: *p* < 0.05, 3rd period: *p* < 0.001). REM sleep was significantly increased at all periods compared to control levels in the GABA or l-theanine alone groups, as well as the GABA/l-theanine mixture group (Figure 4(D)).

NREM sleep consists of *θ* and *δ* waves, with 4.0–8.0 and 0.5–4.0 Hz bandwidths, respectively. The use of l-theanine alone and GABA/l-theanine mixture increased *θ* waves over the ones detected in the control group during all periods. Single l-theanine administration led to a significant decrease in *δ* waves during the 1st period (2.0 h) (*p* < 0.01), which gradually increased in the 3rd period (3.1 h). When GABA/l-theanine mixture was administered, *δ* wave oscillations also gradually increased from 1st (2.2 h) to 3rd period (2.7 h) (Figure 4(E,F)). In conclusion, the combined treatment with GABA and l-theanine led to a significant increase in sleep time (22.4%), especially NREM (28.8%), when administered over a long period. In addition, the use of GABA/l-theanine mixture increased θ wave and decreased δ wave oscillations in NREM sleep, when compared to the control group. Nevertheless, δ waves gradually increased with long-term administration. Therefore, the longer the administration of GABA/l-theanine mixture the better the sleep quality and the longer the sleep duration, induced.

### EEG acquisition and analysis in a caffeine-induced wakefulness model

**Figure 5.**
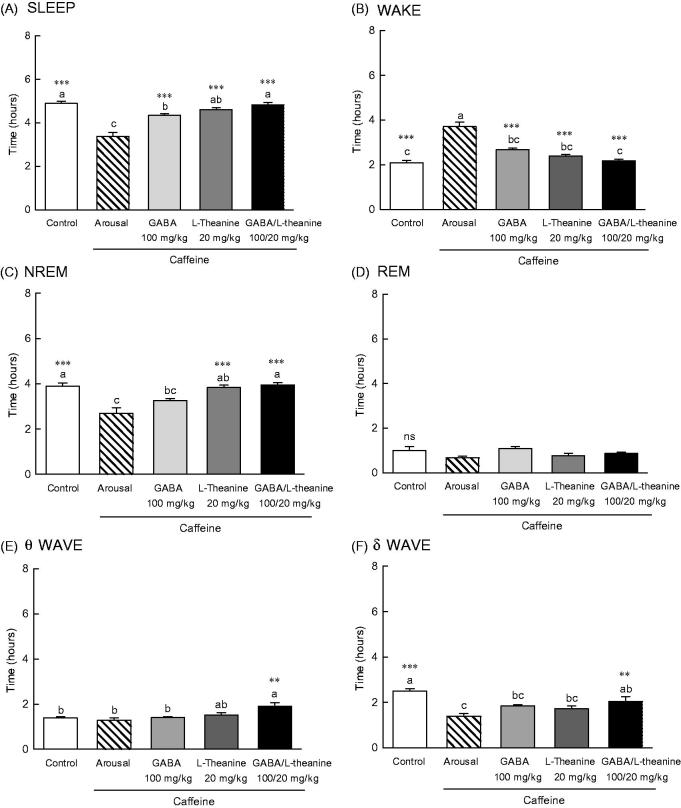
Effects of the GABA, l-theanine, and GABA/l-theanine mixture on caffeine-induced wakefulness in rats at a dosage of caffeine (10 mg/kg). Values are presented as the means ± standard error (SE) for each group, *n* = 8. Different letters indicate significant differences (*p* < 0.05) among samples by Tukey’s multiple range test. Symbols indicate significant differences by Bonferroni test, as ***p < 0.001, **p < 0.01 compared with arousal group.

EEG was performed to assess the sleep-inducing effects of GABA, l-theanine or the combination of both (GABA/l-theanine mixture) in a caffeine-induced awakening animal model (Figure 5). A significant difference (*p* < 0.001) in sleeping and awakening times were observed between the arousal group, which was orally administered 10 mg/kg of caffeine and the control group which was orally administered with saline (Figure 5(A,B)). Administration of GABA, l-theanine, or the combination of both, in the caffeine-induced awakening model led to significant differences in sleep and awakening times, when compared to the arousal group (*p* < 0.001). In the wakefulness model, the use of l-theanine alone or GABA/l-theanine mixture restored NREM sleep time to the control group level (Figure 5(C)). However, the use of GABA/l-theanine mixture in the wakefulness model had a tendency to restore REM sleep time, but there was no significant difference to arousal group. The administration of GABA/l-theanine mixture increased *θ* wave (1.9 h) and increased *δ* wave (2.0 h) oscillations in NREM sleep, when compared to the arousal group (1.4 and 2.5 h, respectively) (Figure 5(E,F), *p* < 0.01). These results suggest that the combined use of GABA and l-theanine rather than GABA or l-theanine alone restores sleep time and quality to normal levels, in the arousal animal model.

### Effects of GABA and l-theanine combination on the mRNA levels of neurotransmitter receptors

**Figure 6.**
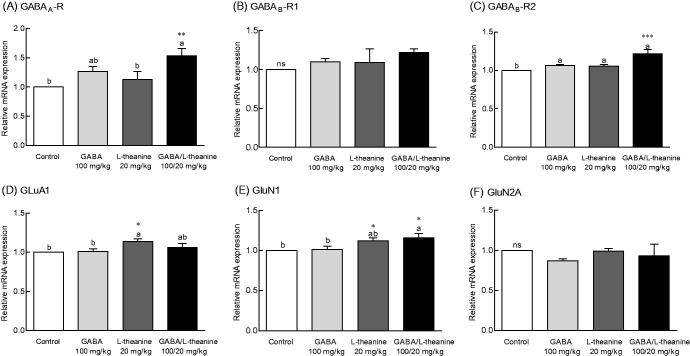
Effects of the GABA/l-theanine mixture on GABA and glutamate receptors mRNA expression in the mouse brain. Values are presented as mean ± standard error of the mean (SEM) for the brain regions of 8 mice and for each group, *n* = 8. Different letters indicate significant differences (*p* < 0.05) among samples by Tukey’s multiple range test. Symbols indicate significant differences by Bonferroni test, as ***p < 0.001, **p < 0.01, *p < 0.05. NS: not significant.

To investigate whether the sleep-promoting mechanism of GABA/l-theanine mixture mediates neurotransmitter receptor expression changes, the mRNA levels of GABA and glutamate receptors were evaluated (Figure 6). Transcript levels for the GABA_A_ receptor following the combined administration of GABA/l-theanine were 1.53-fold higher than control levels (Figure 6(A), *p* < 0.01). Moreover, GABA/l-theanine combined infusion led to significant changes in the mRNA levels of GABA_B_-R2 (21.4%), as compared to controls (Figure 6(C), *p* < 0.001). However, there was no significant difference in the mRNA levels of GABA_B_-R1 compared to the control group (Figure 6(B)).

GABA/l-theanine mixture significantly increased the mRNA levels of the GluN1 glutamate receptor subunit, as compared to control (13.8%) (Figure 6(E), *p* < 0.05). However, administration of l-theanine alone also altered the GluR1 glutamate receptor (12.5%) and GluN1 glutamate subunit (7.2%) expressions when compared to controls (Figure 6(D,E), *p* < 0.05). These results indicate that increased expression of GABA receptors GABA_A_, GABA_B_-R1 and GABA_B_-R2, and the GluN1 glutamate receptor subunit observed with GABA/l-theanine, may possibly lead to improved sleep behaviour and neurological regulation.

## Discussion

The pentobarbital-induced sleeping model was used to identify the optimal sleep enhancement dosing ratio for GABA and l-theanine. In this study, the combination of GABA and l-theanine (100/20 mg/kg) not only did it reduce sleep latency but also prolonged sleep duration in pentobarbital-induced sleep model. The key finding of this study is the confirmation of the synergistic action (*p* < 0.01, compared to control) of GABA and l-theanine on sleep behaviour, which significantly decreased sleep latency and increased sleep duration.

Amino acid neurotransmitters are important for the function of the central nervous system (CNS). They are fast-acting, inducing responses within milliseconds and play an important role in physiological brain function and neurological diseases (Krystal et al. 2002). Rapid neurotransmitter regulation and nerve function, facilitation of relaxation without drowsiness, stress relief (including physical stress), and l-theanine-mediated excitement are known strategies used for the improvement of sleep quality and for exhaustion recovery (Rao et al. 2015).

Sleep deprivation is known to cause serious illnesses such as cardiovascular disease, diabetes, and cancer (Davis and Mirick 2006; Laposky et al. 2008; Baron and Reid 2014). The pentobarbital-induced sleep test and the EEG measurement that have been performed usually in a study of sleep enhancement were used to confirm the synergy effect of GABA/l-theanine mixture in sleep enhancement (Jeon et al. 2015).

The combination of GABA and l-theanine (80/20 and 100/30 mg/kg) did not show a synergistic effect in sleep latency and sleep duration unlike the GABA/l-theanine mixture (100/20 mg/kg). Lin et al. (2010) reported that the synergistic effects were different depending on the ratio of taurine and caffeine, and it is important to identify specific synergistic ratio in the study of combined sample administration. However, further research is needed to determine that synergistic effects are achieved only at a specific ratio with any pharmacokinetic mechanism.

GABA acts through GABA receptors. There are generally 2 types of GABA receptors: GABA_A_ and GABA_B_. The most important receptor, with respect to sleep is the GABA_A_ receptor (Gottesmann 2002). When GABA or another agonist binds to GABA_A_ receptor, it triggers the influx of chloride ions in neuronal cells. This causes a negative membrane potential that inhibits action potential firing. In this way, GABA (and GABA-promoting compounds) reduce activity in brain cells through GABA_A_ receptor activation. It is well-known that the activation of GABA_A_ receptors is beneficial for sleep (Abdou et al. 2006). The structural similarity of l-theanine to the neurotransmitter glutamic acid has prompted researchers to study its potential competition binding on glutamate receptors in the nervous system (Shinozaki and Ishida 1978). l-Theanine rapidly induces changes in serotonergic and dopaminergic transmission (Yokogoshi et al. 1998). These components act as modulating receptors of the neurotransmitter GABA, which is the main inhibitory neurotransmitter in the CNS, and therefore, one of the main molecules responsible for sleeping behaviour (Zanoli and Zavatti 2008). The decreases in sleep latency, together with a slight improvement in sleep quality, are the possible reasons for the observed increase in sleep efficiency, in our study.

As characterized by EEG recordings, sleep is broadly divided into REM and NREM (Bersagliere et al. 2018). Combined oral administration of GABA and l-theanine significantly increased the amount of NREM sleep, as compared to controls (Figure 3, *p* < 0.05), via an increase in theta waves. Moreover, awake time was also significantly decreased following GABA/l-theanine administration, as compared to all other groups (*p* < 0.001, Figure 3). Brain waves can be classified into four types: *α* (less than 8**–**13 Hz), *β* (more than 13 Hz), *θ* (less than 4**–**8 Hz), and *δ* waves (less than 4 Hz) (Abdou et al. 2006). Each wave type is associated with a specific mental state. Delta and theta occur in the early stages of deep sleep and sleep, respectively (Ray and Cole 1985).

We observed a tendency for NREM sleep to increase with increasing dosing periods during the combined oral administration of GABA and l-theanine, probably due to l-theanine (Figure 4). This change in NREM is likely due to changes in delta waves after GABA/l-theanine administration. EEG frequency estimation revealed increased delta and decreased beta activity in the NREM state. Caffeine causes a variety of sleep disturbances, including total sleep time reduction, prolonged sleep onset latency and increased arousal in humans and rats through adenosine receptor blockade (Deckert and Gleiter 1989).

In Figure 5, caffeine decreased sleep time, especially NREM and increased the awake time in rats. The results indicate that GABA and l-theanine combined intake can reverse caffeine-induced sleep reduction, especially NREM, in rats (Figure 5). Administration of l-theanine has been reported to inhibit caffeine’s convulsive action and to increase GABA brain levels in mice (Kimuraand Murata 1971). l-Theanine is known to decrease norepinephrine levels in the rat brain and suppress caffeine-induced serotonin and 5-hydroxyindoleacetic acid increases in rats (Kimura and Murata 1986). Furthermore, the neuroprotective effect of theanine has been shown to be mediated via glutamate receptors, as theanine acts as a glutamate receptor antagonist (Kakuda et al. 2000). The above results suggested that the GABA/l-theanine mixture was significantly superior to GABA or l-theanine alone, for reducing sleep latency, awake time and extending NREM sleep duration. The l-theanine seems to play a major role in the synergistic effect of GABA and l-theanine combination. A trend for prolonged NREM with increasing l-theanine dosing was observed, which was similar to the delta wave increasing trend. In the caffeine-induced arousal model, combined GABA and l-theanine led to a similar synergistic effect on sleep enhancement. The combination of GABA and l-theanine is an attractive NREM sleep-promoting regimen as it increases delta wave oscillations.

As shown in Figure 6, GABA_A_ receptor expression levels were significantly changed in mice with administration GABA**/**l-theanine mixture compared with the control group (*p* < 0.01). The GABA_A_ receptor complex is a chloride ionophore, which consists primarily of GABA, barbiturate, benzodiazepine, steroid and picrotoxin binding sites (MacDonald and Olsen 1994). Parisky et al. (2008) demonstrated that GABA_A_ receptor over expression increases total sleep time, while down-regulation of the receptor decreases sleep duration. The metabotropic GABA_B_ receptor can influence the activation of Ca^+2^ and K^+^ ion channels via G-protein coupled second messengers. The affinity of GABA to GABA_B_ receptors is lower than that for GABA_A_ receptors (Chu et al. 1990). The sedative or sleep-inducing effect of GABA is most likely mediated via GABA receptors. GABA receptor expression in the rat brain was significantly increased following the combined administration of GABA and l-theanine but not after GABA infusion alone. l-Theanine has a similar chemical structure to glutamate and l-theanine has micromolar affinities for kainate, α-amino-3-hydroxy-5-methyl-4-isoxazolepropionic acid (AMPA), and N-methyl-_D_-aspartate (NMDA) glutamate receptors (Kakuda et al. 2002). l-Theanine can act as a competitive glutamate antagonist. The mRNA level of the NDMA receptor subunit GluN1 was slightly higher following GABA/l-theanine infusion than after l-theanine treatment alone (Figure 6).

In conclusion, our results demonstrate that the combined use of GABA and l-theanine increase sleep activity to more than a single administration of either amino acid or these synergistic sleep-promoting effects are likely mediated via changes in GABA and/or glutamate receptor expression in the brain. In summary, this result suggests that GABA/l-theanine mixture could be used for treatment for insomnia and sleep disorders as a concept of nonpharmacological management of sleep.
